# Using a computerized provider order entry system to meet the unique prescribing needs of children: description of an advanced dosing model

**DOI:** 10.1186/1472-6947-11-14

**Published:** 2011-02-21

**Authors:** Jeffrey M Ferranti, Monica M Horvath, Jeanette Jansen, Patricia Schellenberger, Tres Brown, Christopher M DeRienzo, Asif Ahmad

**Affiliations:** 1 Duke Health Technology Solutions, Duke University Health System, Durham, NC, USA; 2 Department of Pediatrics, Duke University School of Medicine, Durham, NC, USA; 3 Duke University Hospital, Durham, NC, USA

## Abstract

**Background:**

It is well known that the information requirements necessary to safely treat children with therapeutic medications cannot be met with the same approaches used in adults. Over a 1-year period, Duke University Hospital engaged in the challenging task of enhancing an established computerized provider order entry (CPOE) system to address the unique medication dosing needs of pediatric patients.

**Methods:**

An advanced dosing model (ADM) was designed to interact with our existing CPOE application to provide decision support enabling complex pediatric dose calculations based on chronological age, gestational age, weight, care area in the hospital, indication, and level of renal impairment. Given that weight is a critical component of medication dosing that may change over time, alerting logic was added to guard against erroneous entry or outdated weight information.

**Results:**

Pediatric CPOE was deployed in a staggered fashion across 6 care areas over a 14-month period. Safeguards to prevent miskeyed values became important in allowing providers the flexibility to override the ADM logic if desired. Methods to guard against over- and under-dosing were added. The modular nature of our model allows us to easily add new dosing scenarios for specialized populations as the pediatric population and formulary change over time.

**Conclusions:**

The medical needs of pediatric patients vary greatly from those of adults, and the information systems that support those needs require tailored approaches to design and implementation. When a single CPOE system is used for both adults and pediatrics, safeguards such as redirection and suppression must be used to protect children from inappropriate adult medication dosing content. Unlike other pediatric dosing systems, our model provides active dosing assistance and dosing process management, not just static dosing advice.

## Background

Medication management in children poses distinctive challenges [1,2], as pediatricians need to calculate doses based on weight, age, gestational age, and indication, which may increase the risk of mathematical errors (such as the serious and common 10-fold overdose) [3]. Pediatric pharmacists often work with adult formulations and must manually compound suspensions for use in pediatric patients. Most importantly, childhood is an inherently dynamic period during which children experience rapidly changing weights and physiologic fluctuations that place them at high risk for incorrect dosing. Given the limited physiologic reserve of pediatric patients, small miscalculations caused by technical glitches or improper system design can cause significant morbidity and mortality [2,4].

Some of these challenges can be addressed using computerized provider order entry (CPOE) systems, and the American Academy of Pediatrics policy statement on the prevention of medication errors strongly recommends the use of "computerized systems" whenever feasible [5]. Research on the epidemiology of adverse drug events (ADEs) in pediatric inpatients reveals that most ADEs originate at the drug ordering stage, with the smallest and most critically ill patients at highest risk [6-9]. In adults, CPOE has been shown to decrease the incidence of medication errors by 55% [10], and CPOE with advanced clinical decision support can decrease error rates by 83% [5,11]. Several studies of pediatric inpatients have demonstrated decreases in medication errors after the implementation of CPOE [8,12,13], and 1 study of pediatric intensive care unit (ICU) patients found a 95% reduction in medication errors and a 40% reduction in potential ADEs following CPOE implementation [14]. However, few studies have shown improvements in post-CPOE implementation outcome measures such as actual (not potential) ADEs, even after meta-analysis of multiple studies [15] or an advanced time-series analysis is performed [16,17]. Furthermore, the landmark study in 2005 by Han reporting an unexpected increase in ICU pediatric mortality related to a pediatric CPOE implementation [18] sparked a nationwide dialogue about the relationship between technology and patient safety. Del Beccaro et al. provided another perspective on this topic by similarly evaluating the same CPOE product in a separate tertiary care facility [19]. They found no mortality increase in critical care areas, and attributed this to a more careful implementation process and the design of ICU-specific order sets. Recently, another group demonstrated a reduction of mortality after implementing a vended but locally modified product at an academic children's hospital [20]. In this time of increased awareness, it is essential that pediatric centers share their experiences to improve large-scale pediatric deployment of CPOE.

Unfortunately, there is significant variability in the level of pediatric-specific medication dosing functionality built into today's CPOE structures. An institution wishing to implement pediatric CPOE often faces a difficult choice between replacing its legacy system with a dedicated pediatric CPOE application [21-23] versus enhancing the existing functionality of an adult-focused CPOE application. At Duke University Hospital (DUH), we chose the latter option, working with our CPOE vendor (Horizon Expert Orders, McKesson Corporation, San Francisco) to adapt our existing, adult-focused system to provide pediatric CPOE. In doing so, we created the "Advanced Dosing Model" (ADM) within the CPOE application to address the unique dosing needs of children. The ADM uses broad clinical decision support to incorporate many criteria into medication dosing such as weight, age, indication, and safety alerts that are built into clinical content. We feel the details of this novel model are sufficiently complex to warrant its own report distinct from other elements of pediatric CPOE.

## Methods

### Setting and implementation period

Duke Children's Hospital (DCH) is a tertiary care facility within DUH that comprises 7 inpatient pediatric service locations: 2 general care medical wards, a pediatric intensive care unit (PICU), a neonatal intensive care unit (NICU), a bone marrow transplant (BMT) unit, a pediatric cardiac ICU, and a transitional care (i.e., step-down) unit. DCH averages 7000 pediatric admissions per year across 187 inpatient beds, approximately 50% of which are located in critical care wards. DCH employs approximately 197 attending physicians and 50 pediatric residents across 20 clinical service areas. Table 1 details release of the CPOE application, which included the ADM functionality described in this report, in order of deployment over a 14-month period across pediatric units (Table 1).

**Table 1:** Deployment of pediatric CPOE at Duke Children's Hospital

Care area	CPOE release date	Current patient beds*
General care (2 units)	1/17/2007	61
Transitional care	1/17/2007	16

PICU	4/24/2007	16
Bone marrow transplant	8/28/2007	16

NICU	3/10/2008	65
Pediatric cardiac ICU^†^	1/06/2009	13

*Number of beds in service as of 1/29/2010.

^†^New pediatric location introduced to DCH after full CPOE deployment on 3/10/2008.

### CPOE architecture at Duke University Hospital

At DUH, the Horizon Expert Orders (HEO) CPOE system (McKesson Corporation, San Francisco, CA) is a comprehensive order management system that spans medical disciplines and offers real-time decision support and guidance for order entry. This product was deployed on all DUH adult floors by April, 2006. Providers interact with a Java-based desktop client (Java version 1.42.09) that queries an Oracle 10 database (Oracle Corporation, Redwood Shores, CA) holding both the clinical content tables (e.g., orderables, order sets, and clinical decision support information) and patient information tables (e.g., patient identity, care area, diagnoses, existing orders). New provider choices made through the CPOE client are saved to the patient information table and executed as HL7 messages to other hospital information technology (IT) applications that fulfill the orders.

### Medication challenges at Duke Children's Hospital

DCH's pre-implementation medication vulnerabilities were similar to those described by other tertiary care institutions and have been reported previously in an analysis of both voluntarily reported events and ADEs detected by computerized surveillance [24]. Briefly, DCH sees approximately 18.0 medication-related safety incidents per 1000 patient days as detected by voluntary reporting. Of these, approximately 10.9% result in some level of patient harm. Computerized surveillance, a complementary incident detection method that behaves as an automated trigger tool, found 1.6 ADEs per 1000 patient days. As expected, event density is higher in critical care units than in general care areas. The most common problem areas are failures in the medication use process such as incorrect drug dose or rate followed by drug omissions. Antibiotics in particular were a drug class identified for enhanced surveillance targeting. We reported that the safety profile of pediatrics was distinct from that of adults, underscoring the importance of pediatric-specific clinical content for dosing guidance [25].

### Needs assessment

Because DCH serves challenging, critically ill pediatric patients, the deployment of CPOE in this environment was deferred until the end of the adult CPOE implementation plan. A needs assessment was performed by a multidisciplinary clinical advisory workgroup of physicians, nurses, pharmacists, and safety directors to define the features and work flow requirements for a pediatric CPOE product deployed to DCH. These individuals made broad decisions that would affect clinician work flow, and their input was critical to ensure operational acceptance. It was immediately recognized that a pediatric CPOE application would require high flexibility to approach the wide variety of nuanced, sometimes novel, pediatric therapies in place at DCH. Given that the existing CPOE system provides adult dosing guidance via a specific set of clinical content tables, it was recognized that the needs of pediatric dosing could be satisfied through adding an additional set of tables for that population while maintaining the existing adult-based infrastructure. As a result, the most efficient plan was to partner with McKesson to enhance the adult product for pediatric usage instead of implementing a new, vended solution. McKesson and DUH agreed to a joint development project to incorporate clinical content from the pediatric WizOrder tool [26], acquired by McKesson from Vanderbilt University Medical Center, into the HEO commercial product. The clinical advisory committee continued to meet weekly for 6 months prior to system release to discuss clinical issues, understand technological implications of the application, and act as liaisons to technical developers at McKesson for all areas of pediatric CPOE design.

### Functional design of the pediatric Advanced Dosing Model (ADM)

By the broadest definition, the ADM as it relates to HEO is a combination of clinical content tables and decision tree logic layered on top of the existing adult CPOE application to provide extensive, content-driven, drug-by-drug clinical decision support for pediatric medication dosing. Based on the clinical advisory committee's recommendations, an ADM focus group was convened to specifically address its functional requirements and design details. This group included 15 pediatric pharmacists charged with defining the detailed patient-specific criteria that shape the dosing scenarios for each drug. To facilitate this, a temporary web application and supporting database was built to store their design decisions and manage the pertinent clinical content.

Instead of simply facilitating weight-based calculations familiar to pediatric dosing, we sought to create a broad clinical decision support system that incorporates up to 6 patient-specific parameters (Table 2) and safety alerts. The major functional design decisions from both the clinical advisory committee and ADM focus group were:

**Table 2:** Pediatric patient parameters reasoned over by the Advanced Dosing Model logic

Criterion	Definition
Indication	A condition that makes a particular medication dose advisable

Care area	Physical location of patient within DCH, which is used to infer care intensity

Chronological age	Age of patient in years, months, and days since date of birth

Post-conceptual age	Age of patient in years, months, and days since clinician-estimated date of conception

Dosing weight	A user-defined weight that will be used to dose medications; this may not reflect a patient's current actual weight

Renal impairment	Qualitative assessment of renal impairment by the ordering provider; i.e., "impaired" or "not impaired"

• The ADM should provide clinical guidance and targeted medication dosing recommendations based on 1 or more patient-centric criteria at the time of order entry.

• The clinical decision support information surrounding medication dosing that populates CPOE clinical content tables should address the diverse needs of general and specialized pediatric populations. In this way, these tables can act as a centralized body of complex yet agreed-upon clinical dosing standards for the hospital.

• The ADM processing logic should be designed to proactively alert clinicians when changes in patient criteria might warrant dosing changes based on the configured clinical knowledge base.

When a single CPOE system is used for both children and adults, the system must differentiate between the 2 populations so that it knows when to implement logic that applies only to children. This is especially important on surgical services where residents care for both adults and children, or in cases of off-service placement where children are cared for on adult floors due to space limitations or unusual practice patterns. After careful multidisciplinary analysis, the advisory group used age solely to define a pediatric patient (< 14 years of age or <18 years and <45 kg [99 lbs]) in order to capture individuals regardless of location.

### Development of pediatric clinical content

To build pediatric clinical content as part of the ADM, unique patient scenarios were identified that may drive a dosing end point for a specific medication. Each unique dosing scenario is termed a medication "dosing region"; that is, the constellation of clinical characteristics that stipulates a specific pediatric dosage for a given drug. A drug that has a variety of clinical usage scenarios therefore has multiple dosing regions. A dosing region can be based on as few or as many criteria as are needed to achieve the specificity required. Similarly, a medication may have numerous associated dosing regions depending on the complexity of the dosing permutations given the patient-specific criteria. For example, Table 3 displays dosing regions built for several ampicillin indications based on different patient scenarios.

**Table 3:** Dosing regions for several ampicillin indications

Indication	Care area	Age	Weight (kg)	Dose
Meningitis	Bone marrow transplant	≤29 days	< 8	100 mg/kg/dose IV q8h
			
			≥8	100 mg/kg/dose IV q8 h up to a max single dose of 1 g
	
	NICU, transitional care	≤7 days	any	100 mg/kg/dose IV/IM q8 h (equivalent to 300 mg/kg/day)
		
		≥8	any	75 mg/kg/dose IV q6 h (approx. 300 mg/kg/day)
	
	PICU, cardiac ICU	≤29 days	≥8	100 mg/kg/dose IV q8 h up to a max single dose of 1 g
			
			Any	100 mg/kg/dose IV q8h

Meningitis or osteomyelitis	General care	≥14 yrs	< 27	75 mg/kg/dose IV q6h
			
			≥27	2 g IV q6h
		
	Non-pediatric location	≤7 days	≥9	100 mg/kg/dose IV q8 h up to a max single dose of 1 g
			
			< 9	100 mg/kg/dose IV q8h
		
		8 days-14 yrs	< 27	75 mg/kg/dose IV q6h
			
			≥27	2 g IV q6h
		
		14-18 yrs	≥27 & <45	2 g IV q6h
			
			< 27	75 mg/kg/dose IV q6h

Pediatric liver transplant - post-op	Any location - dosing region scenarios defined by order set use	≥0 days	< 20	50 mg/kg q6 h × 4 doses
			
			≥20	1 g q6 h × 4 doses

Pediatric liver transplant - pre-op	Any location - dosing region scenarios defined by order set use	≥0 days	< 20	50 mg/kg ONCALLPRN* to OR
			
			≥20	1 g ONCALLPRN* to OR

*The dose is administered once a patient is called to the operating room.

Creating the formulary of adult drugs in the initial launch of adult CPOE was very labor-intensive even when each drug could contain only 1 set of dosing options. We sought to limit initial development to only high-use medications as well as to those that have serious safety concerns. To identify this subset, we pulled information regarding drug utilization for a 1-year period from the pharmacy medication management program. A list including the top 100 most commonly prescribed drugs on pediatric floors was sent to the pediatric pharmacist workgroup. Collective clinical review reduced this list by removing orderables that already had existing clinical decision support through elaborate advisor interfaces (i.e.., insulin prescribing or intravenous fluids). Additionally, chemotherapy is not currently handled within CPOE at Duke University Hospital as it is paper-based and protocol-driven. The workgroup then added medications to this list, regardless of usage frequency, if any of the following conditions were met: a) the drug had high risk of a severe prescribing error; b) the drug had high seasonal usage; or c) the drug appeared on care area "pocket cards." Nurses working in intensive care and BMT units all carry pocket cards that give dosing suggestions for commonly used medications in their care areas. If these medications were located on the card, then they were required to undergo dosing region development. The full drug list was then grouped by American Hospital Formulary Service (AHFS) drug class and prioritized for dosing region development.

Pediatric dosing references [27-29], literature review, and insight from at least 2 clinical pharmacists serving on the ADM task force were used to define dosing regions. Once all dosing regions were designed, a final review was conducted by a sub-specialty physician. By 1 month after ADM deployment, 1200 dosing regions were built for 175 medications. As of July 2009, the knowledge base had expanded to include over 2300 dosing regions for 375 medications. Dosing regions are reviewed and updated based on new clinical evidence once every 2 years.

Because dosing is often based on weights and involves calculation, it was important to create logic that facilitates prescribing drugs in practical amounts. To this end, each dosing region was assigned a rounding method by linkage to a specific data table within the ADM database schema that defined a type of rounding behavior. The "round to 10%" data table lists dosing values where the dispensable dose should be rounded to the closest value in the table, which would ultimately be within 10% of the weight-based calculation. If a calculated dose falls outside of the values in the table, no rounding should occur. A second "round to 5%" data table functions similarly and was put into place for dosing regions requiring tighter control such as those for narcotics, sedatives, and steroids. Finally, specialized rounding tables were created on a drug-by-drug basis to allow for cases where high customization is necessary. For example, several suppository rounding tables were created to reflect commonly dispensable partial suppository amounts for children. The creation of these tables had the effect of forcing selection of drugs in an easily dispensable form.

### Designation of patient parameters describing dosing regions

Each dosing region specifies appropriate dosing advice within the context of 6 patient-specific parameters (Table 2). These parameters were defined by the clinical advisory workgroup discussed previously. One of the more complex issues addressed was patient weight, and it became clear that multiple weights would be required for the system to be clinically relevant to pediatric patients. The concept of "dosing weight" was made distinct from "actual weight" to account for excesses or deficiencies in weight due to fluid imbalances, infections, or feeding issues. Thus dosing weight is the weight on which dosing recommendations should be based, and the clinician should actively decide whether the actual weight is appropriate or a dosing weight should be entered to reflect either transient physiologic changes, such as excess body water, or chronic conditions such as pediatric obesity.

When considering the matter of age for medication dosing, the ADM was designed to provide unit translation for the clinician. In older children, it is appropriate to classify chronological age in whole-number years (e.g., 8 years rather than 8.25 years); however, infants may have age stored in months (or days for newborns). Depending on the age of the child, the ADM may prompt the clinician for gestational age if needed to identify the correct dosing region for a medication.

Renal impairment was included as a potential dosing parameter based upon a qualitative assessment by the ordering provider (i.e., "impaired" or "not impaired"), which is requested when nephrotoxic drugs are dosed. This assessment will result in a suggestion of a different dosage of the drug. Including this as part of the dosing region parameters serves as an important reminder to the ordering provider that the drug is either renally cleared or builds up metabolites and therefore requires consideration of dose adjustment.

The ADM dosing region selection process may require knowledge of a patient's indication. If so, a provider is prompted to select an indication within the CPOE client. Common indication-based dose adjustments include those used in cases of meningitis, osteomyelitis, or an infectious disease. A second pathway to dosing by indication is to use an indication-specific order set for specialized dosing in support of unique disease states or clinical conditions such as sickle cell anemia, cystic fibrosis, or organ transplant dosing. The ADM automatically understands the patient indication based on the order set identity and presents the user with the appropriate, specific spectrum of dosing regions. The user may be prompted further if additional indications are needed to define the dosing region.

### Error prevention measures

When a single CPOE system is used for both children and adults, the system must differentiate between the 2 populations so that it knows when to implement the correct logic. It was recognized early in the planning process that most of the safety features of pediatric CPOE would be undermined if the wrong weight is entered for the patient. Pediatric patients tend to undergo changes in weight more often and to a larger degree than adults, and each manual update of that changing weight is an opportunity for error. To address this, the ADM was configured with several weight-based "pop-up" alerts:

• The user cannot enter any medication without being prompted for a dosing weight if one is not already present. Patient weights are compared to either pediatric or neonatal growth curves stored within the CPOE Oracle database clinical content tables. Users are warned if a patient falls below 3rd percentile or above 97th percentile.

• The user is alerted if there is an extreme change in weight--on average, a change in 10% of the previously recorded value. This threshold is configurable by care area.

• The user is warned if there is an extreme variance between actual and dosing weight, and the variance threshold is configurable by care area.

• If a medication order was dosed off a weight that had since been updated, a reminder to "weight adjust" the medication dose is issued.

• Any patient parameter that is expected to change over time (i.e., dosing weight or actual weight) will raise a less invasive alert (colorful text-based tag next to the variable in question) if it has not been updated after a configurable number of days. It is our expectation that actual weights are updated daily, and dosing weights are updated based on the unit protocol and clinical condition.

Because the pediatric CPOE system was based on a prior adult implementation, adult-specific decision support is left embedded in medication orderables throughout the system. Most orderables contain, for example, adult options for dose, route, frequency, etc., along with instructional text describing appropriate dose recommendations and other considerations specific to that drug. We recognized that it was critically important that pediatricians not be inadvertently presented with adult dosing advice and therefore used a 2-pronged strategy (suppression and redirection) for handling adult-oriented material that may be presented to the end user.

Suppression functionality was implemented for cases where pediatric dosing regions are not available for certain medications but adult-based material is available in the clinical content tables. Any dosing guidance present in the base form of the medication--which is assumed *not *to have been approved for use in pediatrics--is suppressed in the CPOE application code whenever the patient fits our definition of "pediatric." A countermeasure (i.e., a way to "suppress the suppression") was implemented for unique clinical situations where the same dosing principles apply regardless of whether CPOE considers the patient "pediatric" or "adult." The obstetrics service, for example, uses the same labor-and-delivery order sets whether their patient is 12 or 32 years old. Such order sets are designated safe for use in both adults and pediatric populations and thus exempt from the suppressive logic.

Redirection functionality was implemented for cases where it is appropriate for the pediatric prescriber to completely avoid selection of a particular drug. Insulin prescribing, for example, is a complicated endeavor that involves multiple drug orderables because it requires options for different forms of scheduled and supplemental insulin. Clinicians caring for adult patients have always had a full-page, graphical module ("Subcutaneous Insulin Advisor") that presents formal decision support. When the analogous pediatric interface was created, we recognized the risk of a pediatric clinician inadvertently activating the adult-oriented insulin advisor (e.g., with a misdirected mouse-click). Therefore, we created a logic module, triggered by the user's selection of the adult insulin advisor, that determines whether the patient meets the definition of "pediatric" and, if so, redirects the user to the pediatric version.

### Analysis of voluntarily reported safety events

The voluntary Safety Reporting System (SRS) allows staff members to report any perceived safety issues within any DUH care environment, including DCH. DCH staff enters approximately 80 pediatric reports per month. Incidents are reported as being 1 of 9 event categories, and all incidents in the medications category are reviewed by a team of medication safety pharmacists and scored for patient severity. Events of severity of 3 or more (i.e., patient length of stay was increased by the event) are considered ADEs. A full description of the severity algorithm is reported elsewhere [24]. We compared the rate of harmful events (i.e., ADEs) per 1000 patient days and the fraction of total reported events that were ADEs pre- and post- deployment for critical care areas. The Pearson's chi-square test was used to assess statistical significance, and binomial 95% confidence intervals for proportions were calculated.

## Results and Discussion

### Clinician workflow for medication ordering within the CPOE interface

**Figure 1:**
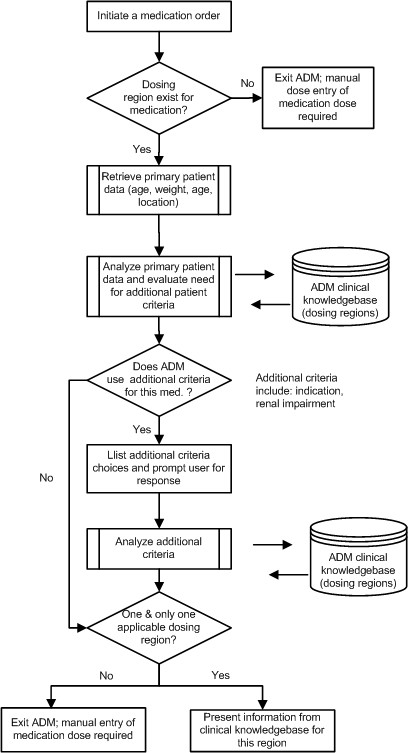
**Processing logic of the Advanced Dosing Model**. A clinician initiates an order for a medication. ADM pre-processing logic verifies if dosing regions exist for this drug. If so, the system will retrieve the available patient information (chronological age, weight, and care area) and prompt the user for any additional information needed. Once all patient parameters are collected, the decision support algorithm will resolve the list of all potential dosing regions from the clinical knowledge base. If the algorithm successfully identifies 1 dosing region, it is presented to the clinician and made available for calculations and screening. If there are no dosing regions available for the requested medication and patient parameters, or multiple dosing regions are found, the system exits the ADM model and the clinician is prompted to enter a medication dose manually.

Users enter the CPOE interface through the organizational electronic health record. The process of ordering medications using CPOE can be initiated by either searching for them using a dialog box within the application or choosing an order set developed for a specific patient profile and then clicking on the hyperlink for an ADM-enabled medication. When a patient is admitted to DCH, details regarding the patient's identity, age, and current physical location are transmitted to the CPOE application from the hospital's admission, discharge, and transfer system. The ADM uses this information, along with prompted provider input and its underlying clinical content tables, to determine whether pediatric medication dosing logic is required (Figure 1).

**Figure 2:**
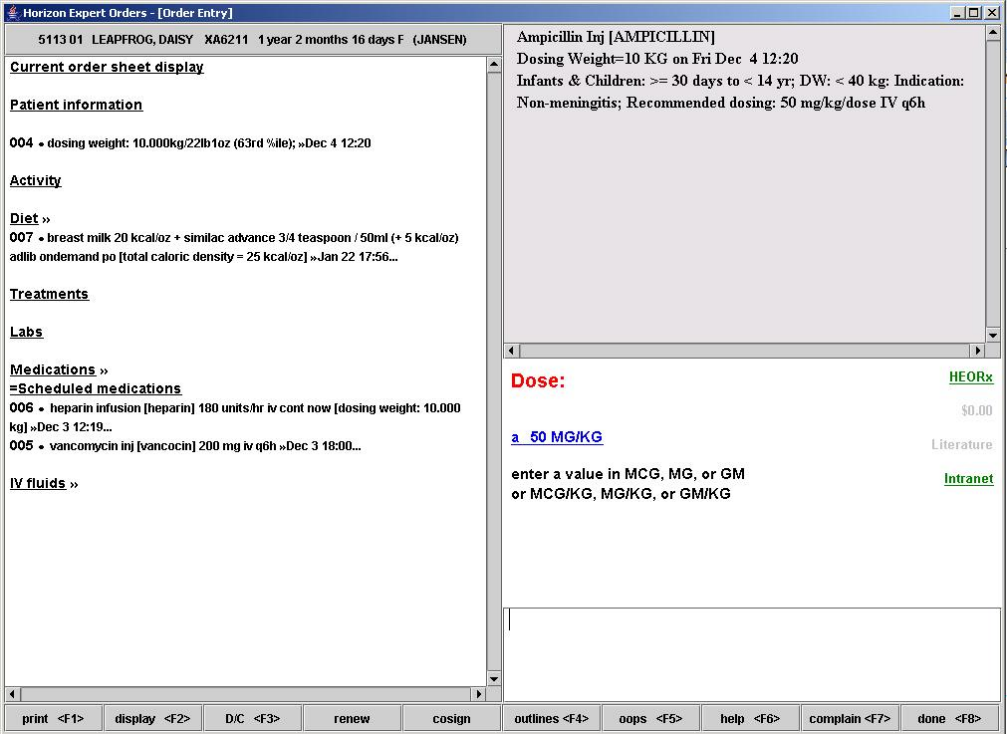
**Screenshot of pediatric medication dosing**. This screenshot shows the presentation of an ampicillin dosing region for an infant at Duke Children's Hospital. The left panel displays the current orders for the patient. The right top panel presents the recommended dosing region based on the patient's dosing weight, age, physical location, and indication. The provider may click and select the suggested value (50 mg/kg) or enter his or her own dosage manually as an override.

Once within the ADM logic, the top half of the screen will display all patient parameters and any relevant considerations or warnings for the drug. The bottom half displays actions to be taken. If an allergy to a medication is known, warnings will be shown at this step, and the user must override the allergy alert twice in the bottom half of the screen before being permitted to move forward with medication ordering. If there are no warnings, the ADM will prompt for any additional information (i.e., indication) and presents the proper dose/frequency combinations determined by the patient parameters. For example, when ampicillin is chosen, the system automatically narrows down the available dosing regions based on the patient's age, weight, and care intensity as defined by location. Because ampicillin dosing regions differ based on indication, it asks the user for this information (e.g., meningitis), and then the system provides the user with appropriate dosing guidance and automatically displays the correct dose and interval to be ordered (Figure 2). During this process, the ADM has calculated dosage based on weight, applied custom numeric rounding, and enforced maximum and minimum doses. Unlike other CPOE applications that present all possible choices of ordering combinations, our application uses the ADM to display a limited subset of recommendations that only includes options deemed appropriate for the patient in question. This greatly reduces display "noise" associated with long lists and helps prevent potential errors due to incorrect selection.

The user can either select this dosing region or manually enter the dosage desired (i.e., an override). From this point, the user will be taken through a series of screens where he or she may enter dose form, start time, duration, and any additional comments for the pharmacy department that are stored in a text field. The last screen will show a summary of the order and all selections made, at which point the user may accept, save as a draft, modify, or exit without saving or ordering. After this terminal step, the order is electronically routed to the pharmacy department for processing. If dosing regions are undefined or do not fit the patient-specific criteria, the system will exit the ADM and prompt the user to manually enter the desired medication order--similar to the process that routinely occurred on paper prior to CPOE deployment.

### Guarding against over- and under-dosing

In allowing providers to override the dosing region logic, it became clear during the testing phase that there is potential for a provider to mistype the dose and order far too much or too little drug. As a result, we programmed extra safeguards into the dosing region knowledge base by having the ADM alert the user if a value is entered that exceeds the minimal or maximal drug doses permitted by any dosing region associated with that medication. To override this, the user must enter the exact "aberrant" value a second time before the ordering process will move forward. We chose this route as opposed to requiring that the user enter a reason for the override to guard against cases where an override is justified and yet an unintentional, excessively extreme dosage is still entered. We believe this level of active participation by the user (as opposed to the oft-used passive alerting; e.g., having to click an "OK" button) strikes a reasonable balance in preventing errors with minimal annoyance. In every case within the CPOE application where alerting methods were used, we recognized that over-alerting (such that the warnings no longer command the user's attention) is as ineffective as no warnings at all and attempted to set alert thresholds accordingly.

### Limited evaluation of CPOE implementation using organizational safety data

Given the extensive literature available that discusses the impact that CPOE may have in terms of unintended patient harm in critical care areas [12,13,15,18,19], we felt compelled to evaluate our intervention using available safety and quality resources. We examined data from our organizational voluntary Safety Reporting System (SRS) to ask whether the rates of adverse drug events increased in pediatric critical care after CPOE deployment. Full details regarding the SRS system have been thoroughly described previously [24,25]. We acknowledge that relying solely upon voluntarily reported events may be problematic due to the well-known issues of reporting bias, volume, seasonality, and anonymity concerns [30]. However, voluntary reporting data have been used elsewhere to evaluate pediatric CPOE systems to better understand the effects of the implementation when a more rigorous prospective study is not possible [12]. Furthermore, SRS is well established within our health system and has become an integral part of the culture of safety at Duke Medicine.

With these caveats in mind, we collected all reported harmful ADEs (i.e., at the minimum, transient adverse patient effects occurred that required some corrective therapy or increased length of stay) [24,25]. The ADE rate decreased 42.9% (p = 0.012) and 46.4% (p = 0.006) in the PICU and NICU units, respectively. Similarly, the percentage of total reports that were severe ADEs decreased significantly in each unit (Table 4). We cannot rule out the effects of reporter bias, and event volume is too low to look at the reports in terms of categories of system failures and attributable causes. Pediatrician review of the event narratives entered by the medication safety pharmacists suggests that there may be fewer PICU reports within 2 primary areas of acknowledged weakness in medication processing--incorrect ordering and order transcription--although the data are sparse. Although we were unable to prospectively study the impact of the advanced dosing model on patient safety, this retrospective analysis of voluntarily reported safety data suggests that we have improved the safety of medication dosing in our pediatric critical care population.

**Table 4:** Voluntarily reported adverse drug events pre- and post-deployment of the pediatric Advanced Dosing Model

Unit	Pre-ADM	Post-ADM
PICU		
No. events	421	410
No. ADEs	53	31
% ADEs (CI)	12.6 (9.4-15.8)	7.5 (5.0-10.1)
% change (p value)	-39.9 (0.016*)	
No. patient days	14,027	14,370
ADEs per 1000 patient days (CI)	3.8 (2.8-4.8)	2.2 (1.4-3.0)
% change (p value)	-42.9 (0.012*)	

NICU		
No. events	567	272
No. ADEs	75	23
% ADEs (CI)	13.2 (10.4-16.0)	8.5 (5.2-11.8)
% change (p value)	-36.1 (0.044*)	
No. patient days	45,627	26,122
ADEs per 1000 patient days (CI)	1.6 (1.2-2.0)	0.9 (0.5-1.3)
% change (p value)	-46.4 (0.006*)	

Pre-period begins on 9/17/2004 and ends the day before the CPOE deployment dates for each unit described in Table 1. Post-period begins the day of CPOE deployment and ends 12/31/2009. CI = confidence interval. *Significant by 2-way Pearson's chi-square test.

### Comparison to other pediatric dosing models

A comprehensive comparison to other systems is hampered by the lack of rigorous reports on CPOE dosing rule development in the formal literature. Commercial CPOE systems, including Horizon Expert Orders, often use formulary references such as First Databank [31] or Lexicon-Multum [32] for static dosing advice (e.g., drug-drug interactions or allergy alerts) but not for active dosing assistance or management of the dosing process where tailored doses are suggested based on the patient's clinical profile. Although true clinical decision support knowledge bases are available, these are focused on adults, require much manipulation for use in the hospital setting, and are underdeveloped for pediatrics [33].

WizOrder, the predecessor of our CPOE system, did evolve to include weight-based dosing [26], but the lack of a full report that describes its implementation is a barrier to comparative analysis. In a 1-page conference proceeding, the authors include the concept of dosing weight, as well as a process by which existing orders are reviewed when the patient's weight changes significantly. Our weight-based dosing also includes checking against growth curves to ensure that the change in weight from the prior value is logical. When 1 new drug is dosed on an updated weight, the other drug orders live on a patient are automatically checked to see if the dosing region is still appropriate or should be updated.

Killelea and colleagues published a description of their pediatric dosing decision support rules for a large teaching hospital [34]. Like our ADM, their method included designing rules by committee based on medication, age, and weight. However, they do not go into much detail regarding how the weights are managed, nor do they describe alerting functionality surrounding weight as we have at DCH. Additionally, their consideration of indication is limited to displaying the dosing guidance for the default indication and providing pop-up windows that describe dosing rules for other less common indications. Patient-specific indication details are not considered by the system, and location-based customization (i.e., care intensity) is not included for presenting a dosing suggestion. Rounding is configurable on a per-medication rule level, but their system as reported is not configurable per location as is the case at DCH.

Our incorporation of location thus comes into focus as an important, novel aspect of our model. We use location as a critical identifier of care intensity, which is especially important given our large BMT and ICU population. These patients often receive augmented doses of medications given the severity of their illness--doses that may be severely harmful to other pediatric patients. It is therefore critical that the decision support inherent to the dosing regions "locks" this content to only the BMT and ICU units. Similarly, cystic fibrosis patients have equally unique medication needs, and so the inclusion of an indication parameter in the ADM allows us to focus clinical content just to this specialized population. Overall, the modular nature of the dosing region model allows us to easily develop new dosing scenarios for specialized populations, especially if the pediatric population profile at DCH changes over time.

### Limitations and lessons learned

The ADM represents a unique, modular approach to manage the logistics of adding increasingly complex clinical decision support information to an existing CPOE application. This allowed us to tailor the adult tool to the unique needs of pediatrics. However, in practice, there are practical and clinical considerations regarding whether a subpopulation warrants specialized dosing using an ADM approach. There can be significant resource management, implementation, and maintenance trade-offs between over-defining and under-defining patient subpopulations. Thus, a core limitation with the dosing region model of medication ordering is that it is not practical to develop a dosing region for every conceivable scenario of pediatric drug dosing. As a result, there will always be certain medications without any dosing regions or instances of dosing region gaps where the ADM is unable to suggest an appropriate dose. Use of dosing regions, therefore, requires that end user clinicians resolve these gaps. In all cases where the ADM is not able to suggest unique dosing, the patient parameters, medications involved, and manually entered dosages are recorded in an audit file for further review to determine if systematic needs are being unmet.

### Addressing patient weight in the provider work flow

Because the ADM relies so heavily on patient weight in pediatric dosing, it became critically important to study the workflow processes that shape how a provider interprets the definition of weight in order to guard against unexpected results. Potential for unintended consequences is significant in a culture that thinks in pounds but doses in kilograms, which would translate into greater than double the intended dose of medication. Providing alerts to address both extremes of the possible weight continuum addresses this issue but has its own challenges. DCH has a large patient population with disease states or chronic conditions that often result in cases of extremely low weight due to poor growth. Even among the general care areas, the increasing prevalence of obese children requires frequent adjustments to commonly accepted dosing paradigms. As a result, crafting alerts that would remind the provider and still avoid alert fatigue was extremely important. Once a patient's weight was entered in the CPOE application, it soon became necessary to define policies surrounding weight definition and responsibilities for its updating during the course of a patient's stay. Although nurses updated the actual weight in their stand-alone documentation system, it was important that this weight was simultaneously updated in the CPOE system. Because of the impact of weight on medication dosing, it was decided that only physicians or physician extenders could enter or update this value.

### Overreliance on technology

One unexpected side effect of the dosing region model was that providers quickly became accustomed to the concept and were concerned when no dosing region was present. Frontline users expected the computer to prevent any bad decisions, and yet, the clinical advisory committee was reluctant to make any restriction on dosing regions that would compromise a provider's flexibility in ordering. Providers had to be educated to think critically about the dosing recommendations for each patient, and were reminded that the thought process in manually entering a dosage drug without a dosing region within CPOE is nearly identical to the prior paper-based ordering process.

### The importance of team composition

Finally, computerized decision support design is not a typical knowledge area for most clinical practitioners, and, conversely, IT system developers often do not possess an understanding of the dynamic health care environment in which their applications are used. Because CPOE is technically sophisticated with immense clinical impact, it is extremely important that the design team include individuals who can bridge the traditional IT and clinical specialty divide. Such individuals review functional and technical specifications with an eye for clinical impact, potential functionality conflicts, and knowledge base gaps.

## Conclusion

In this study, we describe the implementation of a pediatric Advanced Dosing Model that acts as an enhancement to an adult-centric, vended CPOE system in order to meet the unique challenges of pediatric care. Despite some limitations, the ADM provides a powerful way to guide pediatricians through the medication ordering process. The model uses knowledge of the patient's state to deliberate on care parameters and suggest the appropriate dose.

Enhancing an adult-focused CPOE system for safe pediatric medication management is a daunting task. When undertaking such a project, it is essential that physicians and pharmacists with formal informatics training serve as an interface between the clinicians and the development team. We hope that the strategies described here will serve to guide other pediatric institutions as they develop their own plans for the implementation of pediatric computerized provider order entry.

## Competing interests

The authors declare that they have no competing interests.

## Authors' contributions

JMF conceived the project, designed the technical and functional specifications, made critical design decisions, oversaw application deployment, and drafted the manuscript. JJ and PS designed the dosing region functionality and supported frontline deployment. CMD reviewed reported safety incidents and advised on the manuscript. MH performed safety data analysis, interpreted the results, and drafted the final manuscript. AA supported project design and deployment.

## Pre-publication history

The pre-publication history for this paper can be accessed here:

http://www.biomedcentral.com/1472-6947/11/14/prepub
